# Study on Noise Prediction Model and Control Schemes for Substation

**DOI:** 10.1155/2014/696429

**Published:** 2014-02-02

**Authors:** Chuanmin Chen, Yang Gao, Songtao Liu

**Affiliations:** School of Environmental Science & Engineering, North China Electric Power University, Baoding 071003, China

## Abstract

With the government's emphasis on environmental issues of power transmission and transformation project, noise pollution has become a prominent problem now. The noise from the working transformer, reactor, and other electrical equipment in the substation will bring negative effect to the ambient environment. This paper focuses on using acoustic software for the simulation and calculation method to control substation noise. According to the characteristics of the substation noise and the techniques of noise reduction, a substation's acoustic field model was established with the SoundPLAN software to predict the scope of substation noise. On this basis, 4 reasonable noise control schemes were advanced to provide some helpful references for noise control during the new substation's design and construction process. And the feasibility and application effect of these control schemes can be verified by using the method of simulation modeling. The simulation results show that the substation always has the problem of excessive noise at boundary under the conventional measures. The excess noise can be efficiently reduced by taking the corresponding noise reduction methods.

## 1. Introduction

As an important power transfer station, the transformer substation is also the main noise source in power transmission project. It will inevitably affect the station staff, nearby residents, and the local environment. Therefore, the research on substation noise control methods is particularly important. The main noise of the substation comes from working transformer and reactor. The sound of a transformer is a low-frequency noise; it can spread very far and is difficult to eliminate. Though the noise generated by fans and other equipment is mainly high-frequency noise, it is relatively easy to eliminate because high-frequency noise attenuates very quickly. Consequently, it has aroused great attention to seek effective measures to reduce the substation transformer noise.

In recent years, with the development and application of environmental noise simulation software, it provides a new idea for substation noise management. The sound field model of the substation can be established by using the SoundPLAN software. On the one hand, the noise distribution condition of the operational substation as well as the effect of noise on the surroundings can be mastered. On the other hand, reasonable treatment schemes focusing on excessive noise problem can be put forward, and the best noise control method can be obtained by using the simulation function of the SoundPLAN software.

## 2. Analysis on the Transformer Substation Noise

### 2.1. Characteristics of Substation Noise

**Figure 1:**
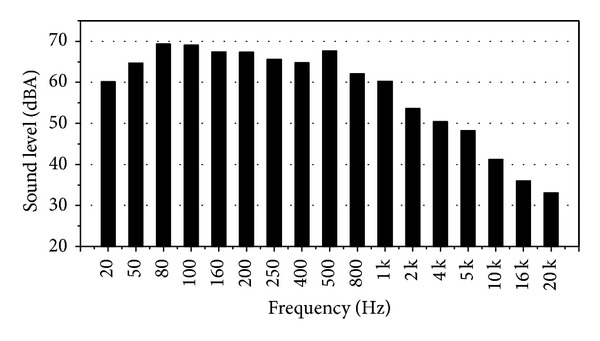
Transformer noise spectrum.

The substation noise mainly comes from the working transformer, reactor, and the cooling system among which transformer is definitely the main noise source. The average noise level of the operating substation is 80~100 dBA [1].  Figure 1 is a transformer noise spectrum of a 330 kV substation in Lanchow, China. As is clearly shown in the spectrogram transformer noise is mainly concentrated in 50~500 Hz frequency band. The whole spectrum mainly shows low-frequency noise. The low-frequency noise has a strong diffraction and transmission capacity. It can easily pass through physical obstacle, so the noise has long-distance transmission and wide range of influence. The low-frequency noise is also the main noise source in residents' daily life. Therefore, the control of the substation noise is mainly focused on low-frequency noise [2].

Substation noise level is increased gradually along with the increase of voltage grade. During the process of ultrahigh voltage substation construction in China, it is not an uncommon phenomenon that the noise exceeds the standard after the project was put into operation [3–7]. Moreover, taking noise control methods in the established substation is more difficult and has a higher cost, while the noise reduction effect is not that efficient. Therefore it is necessary to take noise reduction measures and environmental implication into consideration at the early stage of the substation design process.

### 2.2. Transformer Noise Control Methods

There are three main substation acoustic environment protection measures: noise source control technique, sound insulation technique, and active transformer noise cancellation technique (ATNC). The ATNC technique is making sound offset each other on the same specific frequency by laying up several specific noise generators around the transformer. About 10~20 dBA of noise can be reduced on a certain frequency [8]. While the ATNC technique is complicated and has few application experiences. Noise source control technique can be achieved through the improvement of the production engineering [9]. Experimental results demonstrate that the noise level of transformer depends directly on the magnetostriction size of silicon steel sheet which is used in transformer core. For this reason, the most efficient way to reduce transformer noise is to control and reduce the silicon's magnetostriction by taking effective technical measures. The noise can be reduced about 4~5 dBA by using high quality silicon steel [10, 11], but it will also increase the manufacturing costs. Conventional substation insulation technique is to set up firewalls on both sides of the transformer or the reactor. And the firewalls mainly play a role of sound barriers. With the large-scale substation noise control work commenced, the more frequently used management measures are adding ventilated sound barriers to the transformer and the Box-in technique. The theoretical noise reduction can be 10~15 dBA and 15~20 dBA, respectively [12, 13]. Practice shows that noise barrier has a better performance when the receiver is close to the sound source, while over a long distance it has weaker noise reduction capacity [14, 15]. Box-in technique can effectively reduce the noise at boundary of substation which is equivalent to directly reduce the sound power level of the noise source itself.

### 2.3. Methods to Establish the Substation Acoustic Field Model

**Figure 2:**
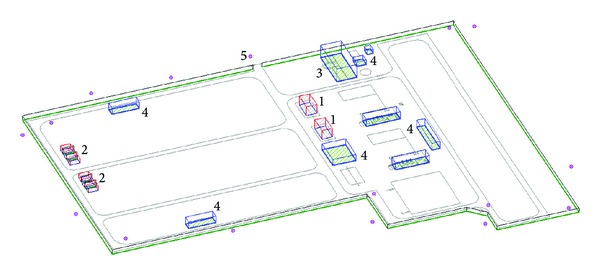
The principal facilities of Lanchow 330 kV substation. 1—330 kV transformer; 2—high voltage reactor; 3—central control building; 4—constructions; 5—noise receiving point.

According to the layout chart of a Lanchow 330 kV substation, the simulation model can be established and acoustic environment simulation can be calculated by using the SoundPLAN software. As shown in Figure 2, the length of substation in latitudinal direction is 260 m, the south width is 197.5 m, and width of the north is 146 m. According to the geometrical shape, the main transformer, reactor is considered as a body sound source which consists of a series of small area sound source. The constructions in the substation are set as the obstacles. A series of noise receiving points were set up beyond substation walls; the distribution of noise at the boundary of the substation can be achieved by putting the sound source data into the model and proceeding simulating calculation process.

### 2.4. Design of Substation Noise Control Scheme

Among the substation noise control measures, the most effective method at present is the Box-in technique. So the design of noise control scheme in this paper is mainly based on the Box-in technique. The Box-in technique is similar to the noise source control measures which are considered to reduce the sound power level of the noise source directly. And its actual amount of noise reduction can reach 10~20 dBA [16]. Four sets of solutions which use Box-in noise reduction technique on transformer and reactor were designed to compare the noise reduction effect of each plan. We choose the amount of 15 dBA noise reduction, which is relatively easy to achieve, to simulate the situation by using Box-in technique. From the actual measured data we can see that the sound power level of transformer is 99 dBA and the sound power level of reactor is 85 dBA. The specific contents of each plan are shown in Table 1.

Based on China [17], if there are residential districts nearby the substation, the noise should be satisfied with the standard of class 2 sound environment functional area, that is, 60 dBA noise emission limits by day and 50 dBA at night.

## 3. Result and Discussion

In the SoundPLAN software, on the basis of substation modeling, the different noise data at the substation's boundary can be achieved by changing the sound source data. Substation is 24 hours of continuous operation, the noise source is stable, and the level of boundary emission noise is relatively consistent at day and night. The attenuation of noise only considers the sound insulation and blocking effect, which is caused by firewalls, bounding walls, and other buildings. The substation generally has hardened cement pavement, so it takes no account of the ground absorption effect and environment greening noise barrier function. And the noise source data of each plan was taken into the SoundPLAN software to make analog computation. The consequences are shown in Table 2.

When there is residential area around the substation, the longest overstandard distance in each plan can be calculated by using the limit standard of 50 dBA. The results are summarized as shown in Table 3.

### 3.1. Plan A

**Figure 3:**
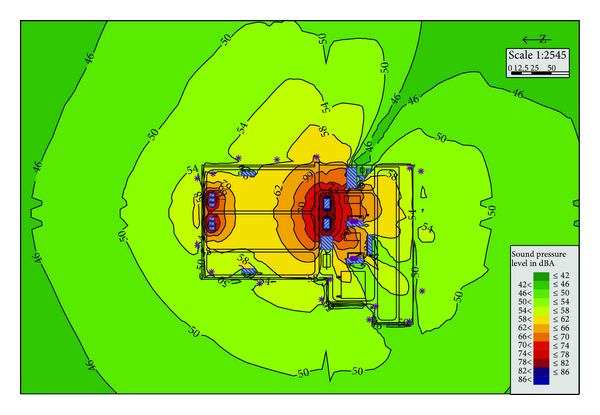
Substation noise distribution in Plan A.

Now the substation generally sets up firewalls on both sides of the transformer or the reactor, so Plan A is the conventional condition of substation. The simulation data in Table 2 shows that noise at the boundary of substation is about 50.9~62.1 dBA. All of the noise at the sound receiving points is above 50 dBA. And it can be seen from Figure 3 that the longest overstandard distance is about 200 m. Compared with other solutions, Plan A has the advantage of less investment and simpler construction. However, after completion of the project, it is required to apply for a wider range of noise control area from the planning department. And also, the use functions of the land around the substation will be restricted.

### 3.2. Plan B

**Figure 4:**
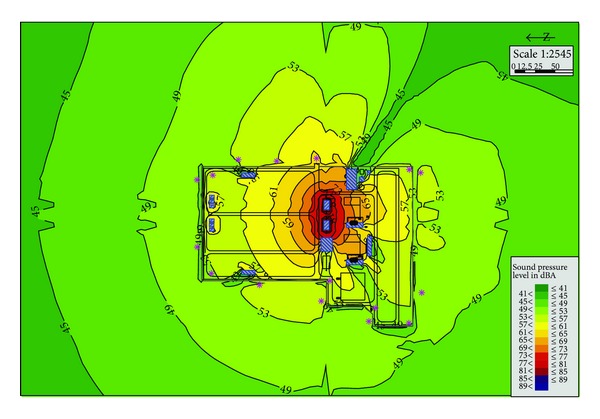
Substation noise distribution in Plan B.

Plan B is based on Plan A, and the reactor adopted Box-in technique, so its sound power level has dropped from 85 dBA to 70 dBA, while the noise of transformer is invariable. After the project is put into operation, the noise at boundary of substation is about 48.8~62 dBA. As can be seen from Figure 4, the noise of the receiving points which located in the north side of the substation and close to the reactor has fallen to below 50 dBA. While the noise in other directions is still a bit large and above 50 dBA; the longest overstandard distance is about 170 m in the east side.

Plan B has been adopted in the ultrahigh voltage substation demonstration project. And its planning noise control scope is smaller than Plan A. It has a certain effect for the acoustic environment protection especially the area near the reactor. But as the noise coming from the transformer has not been reduced, the noise control effect of the whole substation is still not that satisfying.

### 3.3. Plan C

**Figure 5:**
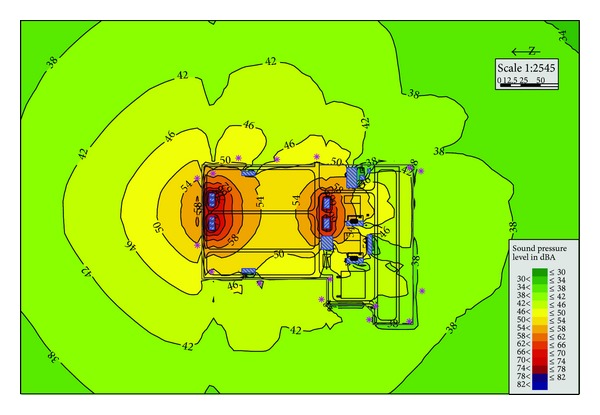
Substation noise distribution in Plan C.

Plan C (Figure 5) is also based on Plan A. The difference is that it adopts Box-in technique on the transformer. As a result, the sound power level of the transformer has dropped from 99 dBA to 84 dBA. The boundary noise is about 38.6~55.5 dBA. The excess noise in the north side of the substation comes from the working reactor because it has no noise control measures. The noise reduction effect is obvious. The longest overstandard distance is about 55 m in the north side.

Because the transformer noise level is greater than the reactor's, when the transformer is adopted in Box-in technique, it has an obvious noise reduction effect. So that most of the boundary noise can meet the class 2 sound environment functional areas requirements in GB12348-2008.

### 3.4. Plan D

**Figure 6:**
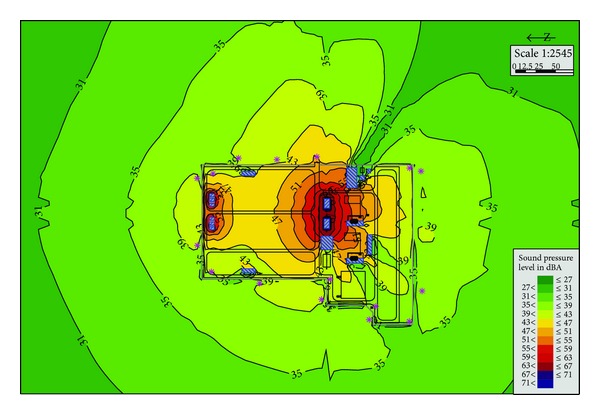
Substation noise distribution in Plan D.

Plan D (Figure 6) is on the basis of Plan B and it applied transformer Box-in technique. This made the transformer noise drop from 99 dBA to 84 dBA, and the reactor noise dropped from 85 dBA to 70 dBA. The boundary noise is about 35.9~47 dBA. The receiving points of the substation in all directions can fully meet the class 2 sound environment functional areas requirements in GB12348-2008. Because in Plan D the main noise sources in the substation have adopted the Box-in technique, this makes the overall noise level of the substation significantly reduced. Now Plan B has been adopted in the HVDC substation projects. This method can efficiently reduce the noise level at the boundary of substation, and it also will not affect the land use functions. It is recommended to be applied in the ultrahigh voltage substation, but it will inevitably increase the construction cost.

## 4. Conclusion

(1) By using the SoundPLAN software, not only can we establish the substation acoustic field model but also we can simulate the noise distribution. The influence of each sound source on the surrounding environment can be intuitively found out by drawing substation noise distribution map. Therefore, using the SoundPLAN software can simulate the substation noise distribution and its surrounding with high accuracy. For this reason, when it comes to building a new transformer substation, environmental noise simulation software like SoundPLAN can be used to make some simulation and calculus during the site selection, design, and construction process. Effective noise reduction measures can prevent the generation of noise as well as limit the spread of sound; it helps to avoid the potential noise problems.

(2) According to conventional design Plan A, the 330 kV substation has a great influence on the acoustic environment. So the use function of land is limited and the sound sensitive areas are influenced. So it is necessary to take a certain amount of noise reduction measures. The effect coming from the operating substation noise mainly depends on some factors such as the voltage level, noise source distribution, and building arrangement. Because the reactor is usually located near the boundary of the substation, though its noise is smaller than the transformer, it is still the main noise source and the focus of noise control.

(3) As for the management of substation noise, the most effective methods are noise source control technique and sound insulation and absorption technology. Among them the prominent Box-in technique can achieve considerable noise reduction level. In Plan B the reactor Box-in technique was used. As a result, the noise level near the reactor has been reduced to about 5 dBA, while on other directions the noise reduction effect is not that obvious. So if the sound sensitive area is close to the reactor, this method is more efficient. When the transformer Box-in technique was used in Plan C, the boundary noise level is mostly below 50 dBA and can meet the standard of class 2 except the north side. The substation can completely meet with class 2 standards for acoustic environmental functional areas just by adding the noise barriers on the north direction.

(4) Using the combination of transformer Box-in and reactor Box-in technique in Plan D, it can significantly reduce the noise level of the whole substation and make the boundary noise meet with the standard of class 1 sound environmental functional area. However, using the Box-in technique will inevitably increase the construction cost, and it also has a certain impact on the cooling performance of the electrical equipment. This solution is suitable for the place where the environmental noise requirement is much higher, for example, educational institution and medical and health organization or other densely populated areas. But from the viewpoint of saving land resources and strengthening environmental protection, using the combination of transformer Box-in and reactor Box-in technique to control the noise of substation is the most optimal method.

## Figures and Tables

**Table 1:** Lanchow 330 kV substation noise control plan.

Items	Constitution	Sound power level before noise reduction	Sound power level after noise reduction	Service condition
Plan A	Firewalls	Transformer 99 dBA Reactor 85 dBA	Transformer 99 dBA Reactor 85 dBA	Conventional design
Plan B	Plan A + reactor Box-in technique	Transformer 99 dBA Reactor 85 dBA	Transformer 99 dBA Reactor 70 dBA	HVDC station and substation have begun to practice
Plan C	Plan A + transformer Box-in technique	Transformer 99 dBA Reactor 85 dBA	Transformer 84 dBA Reactor 85 dBA	HVDC station has begun to practice, substation has not been applied
Plan D	Plan B + transformer Box-in technique	Transformer 99 dBA Reactor 85 dBA	Transformer 84 dBA Reactor 70 dBA	HVDC station has begun to practice, substation has not been applied

HVDC station: high voltage direct current transmission substation.

**Table 2:** Prediction of noise at boundary of Lanchow 330 kV substation dBA.

Noise receiving point	Noise at boundary of substation			
	Plan A	Plan B	Plan C	Plan D
Point 1	62.1	62	48.5	47
Point 2	57.5	53.5	55.5	41.9
Point 3	54.1	52.7	49	38.8
Point 4	56.1	55.9	45.1	41.1
Point 5	52.2	51.9	41.5	37.2
Point 6	51.7	51.5	40.3	36.7
Point 7	55.3	55.3	41	40.3
Point 8	55.7	55.5	45.3	40.7
Point 9	54	53.1	47.4	38.8
Point 10	53.8	48.9	52.4	38.2
Point 11	53.8	48.8	52.3	38.1
Point 12	52.3	52.1	40.4	37.2
Point 13	53.6	53.4	43.2	38.6
Point 14	51.3	51	40.2	36.2
Point 15	50.9	50.8	38.7	35.9
Point 16	52.4	52.4	38.6	37.4

**Table 3:** The overstandard distances of industrial noise by Lanchow 330 kV substation meter.

Direction	The longest overstandard distance			
	Plan A	Plan B	Plan C	Plan D
East	200	170	15	—
South	95	90	—	—
West	115	110	5	—
North	100	80	50	—
